# IMPROVING CAREGIVER ASSESSMENT AND COMMUNICATION ABOUT PAIN IN RELATIVES WITH DEMENTIA

**DOI:** 10.1093/geroni/igac059.2097

**Published:** 2022-12-20

**Authors:** Catherine Riffin, Lilla Brody, Priya Mukhi, Cary Reid, Keela Herr, Karl Pillemer

**Affiliations:** Weill Cornell Medical College, New York, New York, United States; Weill Cornell Medicine, New York, New York, United States; Cornell University, Ithaca, New York, United States; Weill Cornell Medicine, New York City, New York, United States; University of Iowa, Iowa City, Iowa, United States; Cornell University, Ithaca, New York, United States

## Abstract

Pain is under-detected and poorly managed in persons with dementia (PWD). Family caregivers are well situated to detect and facilitate management of pain in PWD, but they receive little guidance and training in these tasks. Our group developed the Pain Identification and Communication Toolkit (PICT), a manualized, multicomponent intervention to help caregivers recognize pain in their care recipients and communicate their observations to healthcare providers. PICT includes a) training in administering an observational pain assessment tool, b) coaching in effective pain communication, and c) building caregivers’ skills through practice. To evaluate PICT’s acceptability, feasibility, and preliminary efficacy, we conducted a pilot randomized controlled trial of N=34 caregivers (n=18 randomized to PICT; n=16 randomized to a control condition). Participants were from diverse racial and ethnic backgrounds (14% Black; 15% Hispanic; 8% Asian; 8% multiracial). Of the caregivers enrolled in the intervention group, 66.7% reported that PICT improved their confidence in identifying pain symptoms, and 83.3% reported that PICT improved their confidence in communicating pain-related concerns to providers. Retention was excellent: 100% of caregivers in the PICT group completed all intervention sessions (4 total); only 5% prematurely terminated the study (did not complete the 12-week post-assessment questionnaire). Notably, caregivers in the PICT group showed a significant improvement in confidence communicating with healthcare providers from baseline to 12-week follow-up (M=3.9 vs. M=4.4; p<.01). Collectively, these findings suggest PICT’s potential as an intervention to help caregivers recognize and communicate about pain in PWD and that a full-scale efficacy trial with larger sample is warranted.

